# Human Milk Oligosaccharide 3′-GL Improves Influenza-Specific Vaccination Responsiveness and Immunity after Deoxynivalenol Exposure in Preclinical Models

**DOI:** 10.3390/nu13093190

**Published:** 2021-09-14

**Authors:** Negisa Seyed Toutounchi, Saskia Braber, Astrid Hogenkamp, Soheil Varasteh, Yang Cai, Tjalling Wehkamp, Sebastian Tims, Thea Leusink-Muis, Ingrid van Ark, Selma Wiertsema, Bernd Stahl, Aletta D. Kraneveld, Johan Garssen, Gert Folkerts, Belinda van’t Land

**Affiliations:** 1 Division of Pharmacology, Faculty of Science, Utrecht Institute for Pharmaceutical Sciences, Utrecht University, 3584 CG Utrecht, The Netherlands; s.seyedtoutounchi@uu.nl (N.S.T.); S.Braber@uu.nl (S.B.); A.Hogenkamp@uu.nl (A.H.); s.varasteh@uu.nl (S.V.); y.cai@uu.nl (Y.C.); A.Leusink@uu.nl (T.L.-M.); I.vanArk@uu.nl (I.v.A.); b.stahl@uu.nl (B.S.); A.D.Kraneveld@uu.nl (A.D.K.); j.garssen@uu.nl (J.G.); G.Folkerts@uu.nl (G.F.); 2 Danone Nutricia Research, 3584 CG Utrecht, The Netherlands; Tjalling.Wehkamp@danone.com (T.W.); sebastian.tims@danone.com (S.T.); selma.wiertsema@danone.com (S.W.); 3 Center of Translational Immunology, Wilhelmina Children’s Hospital, University Medical Center Utrecht, 3584 CG Utrecht, The Netherlands

**Keywords:** mycotoxin, deoxynivalenol, vaccination, immune response, delayed-type hypersensitivity, human milk oligosaccharides

## Abstract

Deoxynivalenol (DON), a highly prevalent mycotoxin food contaminant, is known to have immunotoxic effects. In the current study, the potential of dietary interventions with specific mixtures of trans-galactosyl-oligosaccharides (TOS) to alleviate these effects were assessed in a murine influenza vaccination model. Vaccine-specific immune responses were measured in C57Bl/6JOlaHsd mice fed diets containing DON, TOS or a combination, starting 2 weeks before the first vaccination. The direct effects of TOS and its main oligosaccharide, 3′-galactosyl-lactose (3′-GL), on DON-induced damage were studied in Caco-2 cells, as an in vitro model of the intestinal epithelial barrier. Exposure to DON significantly reduced vaccine-specific immune responses and the percentages of Tbet^+^ Th1 cells and B cells in the spleen. DON significantly altered epithelial structure and integrity in the ileum and reduced the SCFA levels in the cecum. Adding TOS into DON-containing diets significantly improved vaccine-specific immune responses, restored the immune cell balance in the spleen and increased SCFA concentrations in the cecum. Incubating Caco-2 cells with TOS and 3′-GL in vitro further confirmed their protective effects against DON-induced barrier disruption, supporting immune modulation. Overall, dietary intervention with TOS can attenuate the adverse effects of DON on Th1-mediated immune responses and gut homeostasis. These beneficial properties might be linked to the high levels of 3′-GL in TOS.

## 1. Introduction

The mycotoxin deoxynivalenol (DON) is a highly prevalent food contaminant, known to induce immunotoxicity in humans and animals. DON is produced as a secondary metabolite from *Fusarium* fungus species, which contaminates human food at a global level, especially cereal and grain-based products [1,2]. Acute and chronic exposure to DON have significant negative impact on intestinal, neurological and reproductive systems [3]. The immune system is extremely sensitive to DON, since ingestion of very low levels can alter immune responses [4,5]. Depending on the concentration and duration of exposure, both immunosuppressive and immunostimulatory effects can be induced upon DON exposure [6]. Higher doses of DON cause immunosuppressive effects, which may be explained by the apoptosis of leukocytes, whereas immunostimulatory effects are seen after exposure to lower doses [7,8,9]. DON administration in mice decreased the population of antigen-presenting cells and the expression levels of various Toll-like receptors (TLRs) in lymphoid organs, which are critical for immune surveillance [10]. Considering the essential role of the intestinal epithelium in forming a selective barrier between intraluminal dietary antigens and microbes and internal environment, increased gut permeability is associated with different inflammatory diseases and disturbed immune homeostasis [11]. It is already known that DON can damage the intestinal barrier and induce an inflammatory response in vitro and in vivo and increase the gut permeability [12], whereas the addition of specific non-digestible oligosaccharides (NDOs) such as short-chain galacto-oligosaccharides (scGOS) can protect barrier integrity, mainly by facilitating tight junction assembly and reducing the inflammatory response after DON exposure [13].

Specific NDOs can provide prebiotic and immune-modulating properties similar to those observed for human milk oligosaccharides (HMOs). More than two hundred structurally different forms of HMOs have been identified in breast milk [14]; their concentration depends on several factors, including the stage of lactation and the genetic background of the mother [15]. The structural complexity and diversity of HMOs are unique to human milk. They represent the first prebiotics that infants receive and support both microbiome and immune system development. Although NDOs, as well as HMOs, are only partially digested by bacteria in the intestine [16,17], some specific structures, such as 2′-fucosyllactose (2′FL) and galacto-oligosaccharides (GOS), are detectable in the systemic circulation after oral administration [18,19]. HMOs are crucial in the development of a healthy immune system in infants [20]. Various mechanisms have been suggested to explain the immunomodulatory properties of NDOs and HMOs. They are known to be effective prebiotic ingredients and can induce immunomodulatory effects indirectly through microbiota-dependent mechanisms by rebalancing the intestinal microbiota composition and contributing to the development of a healthy intestinal community in infants [21,22,23]. Moreover, HMOs can induce microbiota-independent immunomodulatory effects through direct interaction with immune competent cells [24,25]. Some functional HMO structures, expressed at elevated levels in human colostrum, are based on the elongation of lactose, forming different galactosyl-lactoses (GLs) such as 3′-GL, 4′-GL and 6′-GL [26,27,28]. There are several NDO mixtures, such as short-chain GOS (scGOS) or trans-galacto-oligosaccharides (TOS), which are mainly manufactured via free enzymatic trans-glycosylation or through bacterial fermentation [29], and contain GLs that are identical to those isolated from HMOs [30]. The composition of those NDOs generated by trans-glycosylation highly depends on the enzyme source and technology chosen. Some specific GLs, such as 3′-, 4′- and 6′-GL, may have anti-inflammatory properties on human intestinal epithelial cells in vitro, through inhibition of the NF-κB signaling pathway [27]. Moreover, these GLs have the capacity to attenuate mucosal inflammatory responses during the early developmental stage in intact immature human intestinal mucosa, while supporting the maturation of the intestinal mucosal immune system by modulating Th1/Th2 balance [30]. The beneficial properties of NDOs, improving Th1-dependent vaccine-specific immune responses, have been confirmed in various in vivo studies [24,31,32]. In order to test for possible similar beneficial effects of 3′-GL on the immune system, TOS was used in the present study, which is another NDO mixture high in 3′-GL (Supplementary Figure S1).

Our group has previously reported that DON facilitates allergic sensitization to whey proteins in mice by disturbing intestinal epithelial integrity and inducing cell stress, resulting in an enhanced initiation of Th2 responses and allergic sensitization [33]. However, information about the effect of DON on Th1-mediated immune responses is scarce. As our earlier observations suggest that DON facilitates Th2-dominated immune responses, which are known to inhibit Th1-mediated immunity, we hypothesized that consuming DON can possibly influence vaccine responsiveness in a murine model. A vaccination model is a well-established and validated method for determining the effect of nutritional interventions on cellular and humoral immune responses, with a focus on Th1-mediated responses [34,35]. In addition, the possible immunomodulatory effects of dietary supplementation with TOS, containing high levels of 3′-GL (22% wt/wt), were tested by assessing vaccine responsiveness in DON-exposed mice. Moreover, to understand the possible mechanisms involved, and considering the indispensable role of the gut microbiome on the development of a healthy immune system [36], the changes induced in intestinal epithelial cell layer and microbial activity were investigated both in vivo and in vitro. This study provides key insights regarding the potential of dietary intervention with TOS, attenuating the adverse effects of DON on immunity, by improving Th1-mediated immune responses and re-establishing gut homeostasis.

## 2. Materials and Methods

### 2.1. In Vivo Experiments

Six-week-old female C57Bl/6JOlaHsd mice were purchased from Envigo (Horst, The Netherlands). Upon arrival, mice were conventionally housed with a light/dark cycle of 12 h/12 h (lights on from 7:00 a.m.–7:00 p.m.) at controlled relative humidity (relative humidity of 50–55%) and temperature (21 °C ± 2 °C) with access to food and tap water ad libitum. The animals were randomly grouped with 3 mice per cage in filter-topped Makrolon cages (22 cm × 16 cm × 14 cm, floor area 350 cm^2^, Tecnilab BMI, Someren, The Netherlands) with wood-chip bedding (Tecnilab BMI, Someren, The Netherlands), and tissues (VWR, Amsterdam, The Netherlands) and plastic shelters were available as cage enrichments at the animal facility of Utrecht University. The animals received standard diets (pelleted food, AIN-93G, Ssniff Spezialdiäten, Soest, Germany) and routine care for a week upon arrival in the animal facility, before the start of the experiments. This study was conducted in accordance with institutional guidelines for the care and use of laboratory animals established by the Animal Ethics Committee of the Utrecht University, and all animal procedures related to the purpose of the research were approved under the license of the national competent authority, securing full compliance with European Directive 2010/63/EU for the use of animals for scientific purposes. 

Semi-purified AIN-93G soy protein-based diets were prepared and mixed using different concentrations of DON (FERMENTEK Ltd., Jerusalem, Israel) and/or TOS by Ssniff Spezialdiäten GmbH (Soest, Germany) with concentrations shown in Table 1.

TOS was derived via the transgalactosylation of lactose, according to the previously established methods [37], using *S. thermophilus* ST065 beta galactosidase, after which oligosaccharides were obtained via freeze drying (Danone Nutricia Research, Utrecht, The Netherlands). The dry powder contained β3′-GL (22 g/100 g), lactose (45.5 g/100 g), glucose (15.6 g/100 g), galactose (5.1 g/100 g) and other oligosaccharides (11.7 g/100 g). Using high-pH anion exchange chromatography with pulsed amperometric detection (HPAEC-PAD) the TOS fingerprint was compared to commercial GOS and beta-1,3-galactosyllactose standard (3′-GL, Carbosynth (Berkshire, UK)), as shown in Supplementary Figure S1. 

#### 2.1.1. Study Design

**Figure 1:**
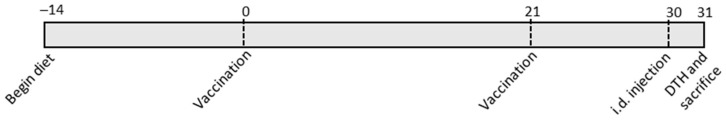
A schematic overview of the murine model of vaccination.

After a week of acclimatization, animals were randomly divided into 10 groups (Table 1) and received either control or modified diets. A schematic overview of the experimental design is shown in Figure 1. Vaccination was conducted 2 weeks after starting the diets, using Influvac (Abbott Biologicals B.V., Weesp, The Netherlands) from the 2015/2016 season, as previously described [38]. The mice received the primary and booster vaccinations via subcutaneous injections of 100 µL undiluted Influvac (containing hemagglutinin (HA) and neuraminidase antigens of three strains of myxovirus influenza, in a dose equivalent to 30 µg/mL HA per strain, in total 90 µg/mL HA) in a skin fold of the neck. The booster vaccination was given 21 days after the primary vaccination. The sham-treated group (*n* = 3, negative control) received injections of 100 µL PBS instead of the vaccine in order to demonstrate the specificity of the vaccine-induced response.

The animals were weighed before starting the diets (day 14) and before booster vaccination (day 21). The weight gain was calculated using the formula:

(1) (weight on day 21)−(weight on day −14)=weight gain (g)

#### 2.1.2. Antigen-Specific Delayed-Type Hypersensitivity Reactions

Delayed-type hypersensitivity (DTH) reactions were induced 9 days after booster vaccination via an intradermal (i.d.) injection of 20 µL Influvac into the ear pinnae of the right ear, and 20 µL PBS into the ear pinnae of left ears as basal line, under isoflurane-induced anesthesia. Ear thickness was measured in duplicate using a digital-micrometer (Mitutoyo Digimatic 293561, Veenendaal, The Netherlands) before injection and 24 h thereafter. The antigen-specific DTH responses were calculated using the formula:

(2) (right ear (thickness at 24 h−thickness at 0 h))−(left ear (thickness at 24 h−thickness at 0 h)=DTH (µm)

The measurements were performed in randomized order and blinded.

#### 2.1.3. Vaccine-Specific Immunoglobulins (Igs) in Serum 

Blood was collected at the end of the experiment through orbital extraction under inhalation anesthesia induced by isoflurane, and then cervical dislocation was applied. Blood samples were centrifuged (10,000 rpm for 10 min) to collect the serum and were stored at −20 °C until analysis. To determine serum concentration of vaccine-specific antibodies, the enzyme-linked immunosorbent assay (ELISA) was performed as described previously [39]. Serum samples were incubated in 96-well plates (Costar EIA/RIA plate, Alphen a/d Rijn, The Netherlands) pre-coated with 1:100 diluted Influvac in PBS. Final dilutions of 1:2000 and 1:8000 of serum samples were used for IgG1 and IgG2a measurements, respectively, and a dilution series of pooled serum that contained vaccine-specific antibodies was added for standard curve calculation [38]. For blocking nonspecific binding, the plate was incubated for 1 h with 2% BSA (Sigma, Zwijndrecht, The Netherlands) in PBS at room temperature. Anti-IgG1-biotin and anti-IgG2a-biotin (Becton Dickinson, Heerhugowaard, The Netherlands) antibodies were diluted 1:1000 in dilution buffer (PBS with 0.5% BSA and 0.1% Tween). The plates were subsequently incubated with a 1:20,000 dilution of streptavidin-HRP (Biosource, Etten-Leur, The Netherlands) and optical density was measured with a Benchmark microplate reader (BioRad, Hercules, CA, USA) at a wavelength of 490 nm. Concentrations in test sera were calculated in arbitrary units (AU), relative to the standard curve.

#### 2.1.4. Splenocyte Isolation and Flow Cytometry of Immune Cells

Fresh splenocytes were isolated from spleens using methods described previously [39]. After removing red blood cells by incubating them in lysis buffer (8.3 g NH_4_Cl, 1 g KHC_3_O, and 37.2 mg EDTA dissolved in 1 L demi water and filter sterilized), splenocytes were counted and resuspended in RPMI 1640 medium containing 10% fetal bovine serum and penicillin (100 U/mL)/streptomycin (100 µg/mL) to reach the concentration of 10^7^ cell/mL. Cells were washed in PBS/1% BSA and incubated with anti-mouse CD16/CD32 (1:100 dilution in PBS/5% BSA; Mouse BD Fc Block, BD Pharmingen, San Jose, CA, USA) to block non-specific binding sites. For surface staining, cells were incubated at room temperature for 1 h with CD4 Brilliant Violet 510, CCR6-PE (BioLegend, San Diego, CA, USA), CD69-PE-Cy7, CXCR3-PE, CD25-PerCP-Cy5.5, (eBiosciences, Thermo Fisher Scientific, San Diego, CA, USA) and T1ST2-FITC (MD Biosciences, St. Paul, MN, USA). Viable cells were distinguished by means of a fixable viability dye, eFluor^®^ 780 (eBioscience). For the detection of intracellular transcription factors, cells were first fixed and permeabilized with the Foxp3 Staining Buffer Set (eBioscience) according to the manufacturer’s protocol and then stained with Foxp3-FITC (eBioscience), GATA3- PerCP-eFluor710 (eBiosciences, Thermo Fisher Scientific) and Tbet- Alexa Fluor647 (BioLegend) antibodies. Details of the antibodies, including RRID and dilution, are shown in Supplementary Table S1. Results were collected with a BD FACSCanto II flow cytometer (Becton Dickinson, Franklin Lakes, NJ, USA) and analyzed with FlowLogic software (Inivai Technologies, Mentone, VIC, Australia).

#### 2.1.5. Re-Stimulation of Splenocytes with Vaccine-Loaded Bone Marrow-Derived Dendritic Cells (BMDCs)

Bone marrow cells were isolated from the femurs and tibias of healthy 11-week-old C57BL/6JOlaHsd mice, and were cultured in RPMI 1640 medium (Gibco) supplemented with 10% FBS and 100 U/mL penicillin/streptomycin, 10 mM HEPES, 1 mM sodium pyruvate, and Eagle’s minimum essential medium (MEM) non-essential amino acids (all from Gibco Life Technologies) in the presence of 10 ng/mL GM-CSF (Prosepec, The Netherlands) for 6 days to obtain immature BMDC (iDC) [38]. Induced iDCs were then loaded with Influvac vaccine at a concentration of 0.9 µg/mL and incubated for 24 h at 37 °C, 5% CO_2_ to obtain matured DCs. iDCs treated with medium were used as negative controls. Splenocytes collected from vaccinated mice were cocultured with matured DCs at a 10:1 ratio, in 96-well U-bottom culture plates for 5 days at 37 °C and 5% CO_2_, with supplemented RPMI 1640 medium (Gibco).

Cell culture supernatants were collected at day 5 and stored at −20 °C until use and analyzed for the concentration of interleukin (IL)-4, IL-6, IL-10, IL-13, tumor necrosis factor (TNF)-α, macrophage inflammatory protein (MIP)-2 and interferon (IFN)-γ using a ProcartaPlex multiplex protein assay kit (Invitrogen, Thermo Fisher Scientific, Waltham, MA, USA) according to the manufacturer’s instructions.

#### 2.1.6. Isolation of RNA from Intestinal Samples for qRT-PCR

For mRNA isolation, ileum samples (1 cm before the ileocecal junction) collected from vaccinated mice were immediately frozen on dry ice and kept at −80 °C until analysis. Tissue samples were weighed and homogenized in lysis buffer containing β-mercaptoethanol (provided within the RNA isolation kit) with a 1:1 (*w*/*v*) ratio, as described before [40]. Total RNA was isolated using spin columns based on the manufacturer’s instructions (SV Total RNA Isolation System, Promega Corporation, Madison, WI, USA) and RNA content was measured using the NanoDrop ND-1000 Spectrophotometer (Thermo Fisher Scientific, Wilmington, DE, USA). The RNA purity of the samples was confirmed by calculating the 260/280 nm and 260/230 nm ratios. Subsequently, the iScript cDNA Synthesis kit (Bio-Rad Laboratories, Hercules, CA, USA) was used to reverse-transcribe the RNA into cDNA, using the T100 thermal cycler (Bio-Rad Laboratories, Hercules, CA, USA).

For qPCR, the reaction mixture was prepared by adding selected primers (Bio-Rad Laboratories, Hercules, CA, USA) and iQSYBR Green Supermix (Bio-Rad Laboratories, Hercules, CA, USA) to the samples, and amplifications were performed according to the manufacturer’s instructions using the CFX96 Touch™ Real-Time PCR Detection System (Bio-Rad Laboratories, Hercules, CA, USA). The mRNA for each gene was normalized to the expression of GAPDH as a reference, and the relative mRNA expression for each mouse was depicted as a fold change of the average of the control group.

#### 2.1.7. Histomorphometric Analysis of Intestinal Specimens

For histomorphometric analysis, ileum samples collected from vaccinated mice were fixed in 10% neutral buffered formalin in the form of Swiss rolls and embedded in paraffin. The 5-μm sections were stained with hematoxylin/eosin according to standard procedures [41]. Photomicrographs were taken with an Olympus BX50 microscope equipped with a Leica DFC 320 digital camera (magnification of 200×). The morphometric analysis of the sections was performed on 10 randomly selected, well-oriented villi and crypts per animal. A computerized microscope-based image analyzer (Image Pro MC) was used to determine villus height (measured from the tip of the villus to the villus–crypt junction) The villus height was manually defined for each villus separately [12].

#### 2.1.8. Short-Chain Fatty Acid (SCFA) Concentrations in Cecum

Cecum content was collected and immediately frozen on dry ice and stored at −80 °C until analysis. The samples were homogenized in cold PBS and clear supernatants of the homogenates were collected after centrifuging and were stored at −80 °C until analysis. For the measuring of the concentration of acetic, propionic, and butyric acids by gas chromatography, as previously described [42], we used 2-ethylbutyric acid as an internal standard.

### 2.2. In Vitro Experiments

Human epithelial colorectal adenocarcinoma (Caco-2) cells were obtained from American Type Tissue Collection (code HTB-37, Manasse, VA, USA, passage 90–102). Beta-1,3-galactosyllactose (β3′-GL) was obtained from Carbosynth (Berkshire, UK) and alpha-1,3-galactosyl-lactose (α3′-GL) was obtained from Elicityl (Crolles, France). Purified deoxydivalenol (DON) (Sigma Aldrich, St Luis, MO, USA) was dissolved in pure ethanol and stored at −20 °C. TOS was obtained as described above by freeze-drying the sugar products of *S. thermophilus* ST065 beta galactosidase (Danone Nutricia research, Utrecht, The Netherlands).

#### 2.2.1. Cell Culture

Caco-2 cell monolayers were grown in a trans-well system, which is a model for studying intestinal barrier function [43]. Dulbecco’s modified Eagle’s medium (DMEM; Gibco, Invitrogen, Carlsbad, CA, USA) with 10% fetal bovine serum (FBS) and 100 U/mL penicillin/streptomycin was used as culture medium and cells were seeded at a density of 0.3 × 10^5^ cells into 0.3 cm^2^ high pore density (0.4 µm) inserts with a polyethylene terephthalate membrane (BD Biosciences, Franklin Lakes, NJ, USA) placed in a 24-well plate. The Caco-2 cells were maintained in a humidified atmosphere of 95% air and 5% CO_2_ at 37 °C. After differentiation of intestinal epithelial cells measured by transepithelial electrical resistance (TEER) exceeding 400 Ω cm^2^ (Millicell Electrical Resistance System volt-ohm-meter, Millipore, Temecula, CA, USA), the interventions were initiated. The monolayers were then pretreated for 24 h with 0.25% or 0.5% 3′-GL, or with TOS at the concentration range of 0.25% to 1% before being exposed to DON, which is known to impair intestinal barrier [12]. Subsequently, DON was used at a concentration of 4.2 µM in complete cell medium and added to the apical side, as well as to the basolateral side of the trans-well inserts for 24 h.

The effect of DON, TOS and 3′-GL on cell viability at concentrations used in this study was evaluated by measuring LDH leakage in the culture medium using the Cyto-Tox 96 nonradioactive cytotoxicity assay kit (Promega Corp., Madison, WI, USA), according to manufacturer’s instructions.

#### 2.2.2. Caco-2 Cell Monolayer Integrity Measurement

Measurements of the TEER and lucifer yellow (LY) permeability were conducted to investigate intestinal barrier integrity. TEER measurements were performed as previously described [12] and results are expressed as % of the initial value, as measured prior to the pretreatment.

For the determination of paracellular transport, the membrane-impermeable lucifer yellow (LY) (Sigma, St Luis, MO, USA) was added to a concentration of 16 µg/mL to the apical compartment in the trans-well plate for 4 h, and the paracellular flux was determined by measuring the fluorescence intensity in the basolateral compartment with a spectrophotofluorimeter (FLUOstar Optima, BMG Labtech, Offenburg, Germany) set at excitation and emission wavelengths of 410 and 520 nm, respectively.

#### 2.2.3. Cytokine Production

Within cell culture supernatants, the release of interleukin-8 (IL-8 or CXCL8), which is a typical marker for inflammation, was quantified in the medium of the apical side and the basolateral side of the Caco-2 trans-well inserts in response to the treatments. CXCL8 concentrations were measured by using the human ELISA assay (BD Biosciences, Pharmingem, San Diego, CA, USA) according to the manufacturer’s instructions.

## 3. Statistical Analysis

All data were analyzed by GraphPad Prism 8.0 software (GraphPad Software, San Diego, CA, USA) using one-way ANOVA, followed by Bonferroni’s multiple comparison post hoc test for selected comparisons, and for non-normally distributed and non-parametric data, the Kruskal–Wallis test was performed, followed by Dunn’s multiple comparisons test. Data are presented as mean ± SEM. * *p* < 0.05, ** *p* < 0.01 and *** *p* < 0.001 were considered statistically significant.

The required sample size for in vivo studies was calculated based on the DTH data available from previous vaccination experiments [38], using G*Power v3.1.9. The estimated effect size was calculated as 0.65 based on our previous experiments, where an outcome of 10% change in ear thickness is considered the minimum relevant difference. The power was set at 0.9 and α was corrected for the number of relevant comparisons (Supplementary Table S2).

## 4. Results

### 4.1. In Vivo Results

#### 4.1.1. Dietary TOS Improved Vaccine-Specific Cellular and Humoral Responses in DON-Exposed Mice

**Figure 2:**
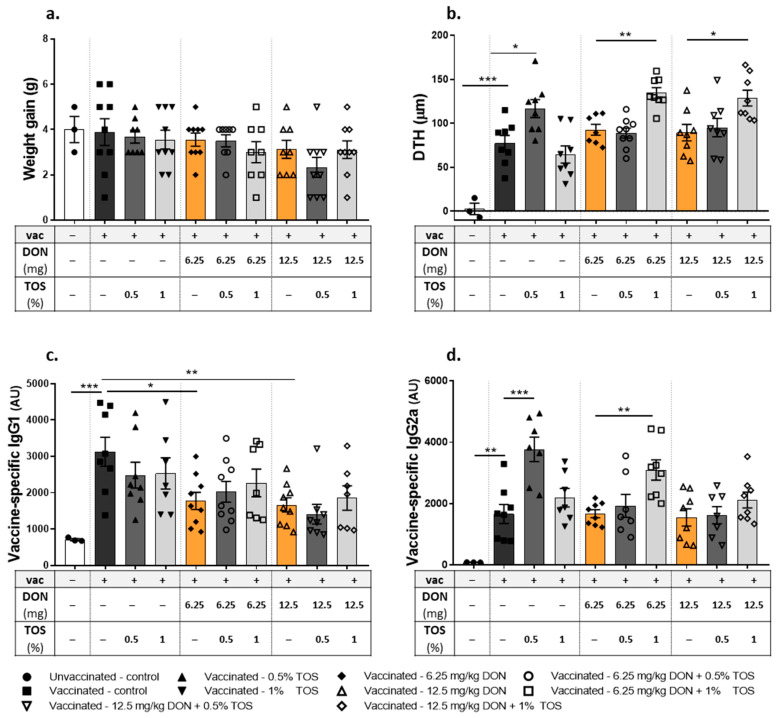
Effect of dietary intervention with deoxynivalenol (DON) and trans-galacto-oligosaccharides (TOS) on weight gain, vaccine-specific delayed-type hypersensitivity (DTH) responses and antibody levels in serum collected on day 31 from vaccinated (vac +) and unvaccinated (vac −) mice. (**a**) Weight gain throughout the experiment; (**b**) DTH response; (**c**) vaccine-specific IgG1; and (**d**) IgG2a levels in serum, measured by means of ELISA assays. Data are presented as mean ± SEM. * *p* < 0.05, ** *p* < 0.01 and *** *p* < 0.001 indicate statistical differences.

There was no significant difference in average weight gain between different dietary groups (Figure 2a). There was a significant antigen-specific response to Influvac after i.d. injection, as determined by assessing the DTH reaction (measured as ear swelling) and vaccine-specific IgG1 and IgG2a levels in the serum of mice fed with the control diet. Interestingly, a significant (*p* < 0.05) difference in the influenza-specific DTH response was detected in mice receiving 0.5% TOS as compared to vaccinated mice receiving the control diet (Figure 2b). Dietary DON contamination induced no significant effect on the DTH response, compared to the vaccinated control group. However, when TOS was added to DON-contaminated diets, the higher concentration of TOS (1%) was required to induce significant vaccine-specific immune boosting effects (Figure 2b).

Vaccine-specific IgG2a and IgG1-antibody concentrations were elevated in all vaccinated mice compared to the sham group. TOS supplementation had no significant effect on the DTH-induced increase in IgG1, but it significantly increased (*p* < 0.001) the serum level of IgG2a at the concentration of 0.5% (Figure 2c,d) in vaccinated mice. Dietary DON contamination induced a significant reduction in the IgG1 level in vaccinated mice (*p* < 0.05 and *p* < 0.01 for the concentrations of 6.25 mg DON/kg and 12.5 mg DON/kg, respectively), whereas it had no effect on enhanced IgG2a level. The addition of TOS to DON-contaminated diets had no effect on the DON-induced reduction in IgG1 production, but it was able to significantly increase the IgG2a level (*p* < 0.01) in animals receiving 6.25 mg DON/kg in their diet (Figure 2c,d).

#### 4.1.2. Dietary TOS Increased Th1 Cell Activation and Attenuated DON-Induced Modification in T Cell Populations in the Spleen

**Figure 3:**
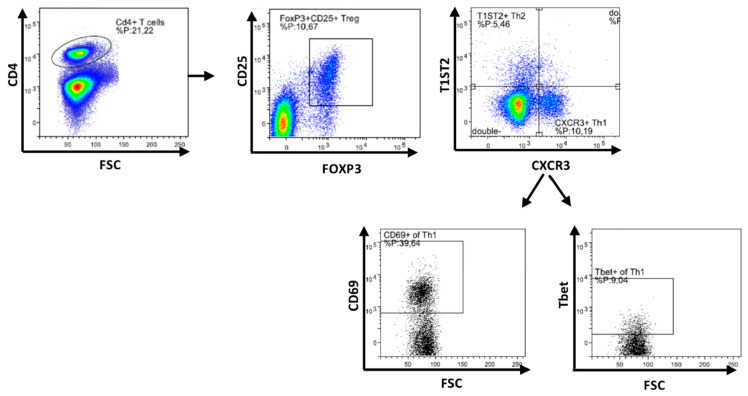
Flow cytometric analysis of T cell subpopulations in spleen. Gating strategy for selecting CD25^+^Foxp3^+^ regulatory T cell (Treg), T1ST2^+^ Th2 cells, CXCR3^+^ Th1 cells and CD69^+^ and Tbet^+^ cells from CXCR3^+^ Th1 cells.

**Figure 4:**
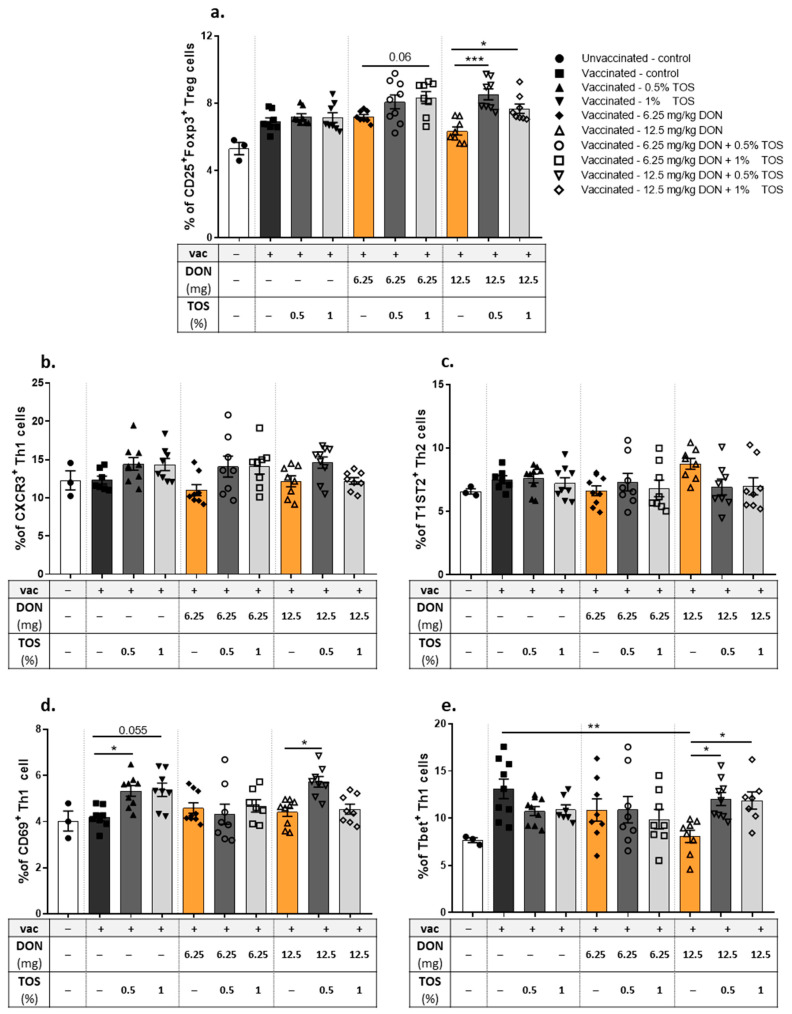
Flow cytometric analysis of T cell subpopulations in the spleen of vaccinated (vac +) and unvaccinated (vac −) mice fed on diets containing deoxynivalenol (DON) and/or trans-galacto-oligosaccharides (TOS). Percentages of (**a**) CD25^+^Foxp3^+^ regulatory T cell (Treg), (**b**) CXCR3^+^ Th1 cells, (**c**) T1ST2^+^ Th2, (**d**) CD69^+^ CXCR3^+^ Th1 cells and (**e**) Tbet^+^ CXCR3^+^ Th1 cells. Data are presented as mean ± SEM. * *p* < 0.05, ** *p* < 0.01 and *** *p* < 0.001 indicate statistical differences.

The frequency and activation status of regulatory T cells (Treg) and T helper cells (Th1 and Th2) in isolated spleen samples were studied using flow cytometry (Figure 3, gating strategy). The vaccine-induced DTH reaction did not significantly affect the percentage of CD25^+^ FoxP3^+^ Treg cells in the spleen (Figure 4a). In addition, the percentage of these Treg cells was not significantly affected by the addition of TOS or DON into the diet of vaccinated mice, but the addition of TOS to DON-contaminated diets significantly increased the percentage of Treg cells in the spleens of vaccinated mice, compared to the animals exposed only to DON (Figure 4a).

The vaccine-induced DTH reaction did not significantly affect CXCR3^+^ Th1 cell and T1ST2^+^ Th2 cell percentages in the spleen (Figure 4b,c). In addition, no significant effect due to the presence of TOS or DON in the diet was observed on the levels of CXCR3^+^ Th1 cells or T1ST2^+^ Th2 cells in vaccinated mice. However, both concentrations of TOS increased CD69^+^ activated Th1 cell numbers compared to those in vaccinated animals in control diet group (*p* < 0.05) (Figure 4d). DON contamination with the higher concentration used in this study induced a significant reduction in Tbet^+^ CXCR3^+^ Th1 cells in the spleen of vaccinated mice, compared to controls (*p* < 0.01), but the addition of TOS with both concentrations increased the percentage of Tbet^+^ Th1 cells in the spleen of DON-exposed mice (*p* < 0.05), and restored it to the values of the control group (Figure 4e).

#### 4.1.3. Dietary TOS Reversed the Effect of DON on the B Cell Population and Activation in the Spleen

**Figure 5:**
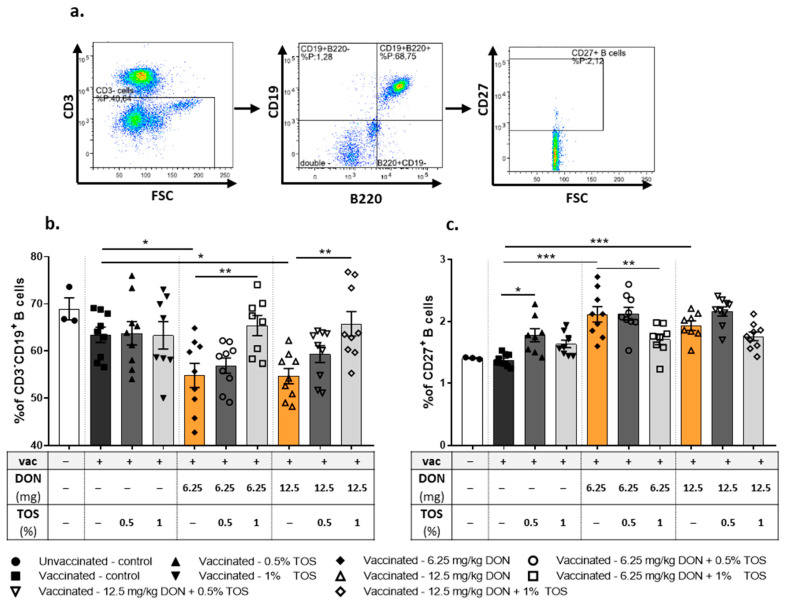
Flow cytometric analysis of B cell subpopulations in spleen of vaccinated (vac +) and unvaccinated (vac −) mice fed on diets containing deoxynivalenol (DON) and/or trans-galacto-oligosaccharides (TOS). Gating strategy (**a**), percentage of CD19^+^B220^+^ B-cells (**b**) and CD27^+^ memory B cells (**c**). Data are presented as mean ± SEM. * *p* < 0.05, ** *p* < 0.01 and *** *p* < 0.001 indicate statistical differences.

The frequency and activation status of B cells in isolated spleen samples were studied using flow cytometry (Figure 5a, gating strategy). Surface marker expression analysis of CD19 and CD220 revealed no significant effect on CD19^+^ B220^+^ B cells in the spleens of vaccinated and control-diet- or TOS-receiving mice compared to the control mice (Figure 4). Both concentrations of DON induced significant reductions in CD19^+^ B220^+^ B cell populations in the spleens of vaccinated mice undergoing a DTH reaction (*p* < 0.01). In line with the changes detected in the IgG level, the addition of 1% TOS to DON-contaminated diets significantly (*p* < 0.001) increased the percentage of B cells in the spleen and was able to restore the effect of DON on the DTH-induced B cell response in the spleen (Figure 5b).

CD27 expression was used to distinguish between memory and naive B cells [44]. The DTH reaction had no significant effect on the percentage of memory B cells. Both concentrations of TOS significantly increased the percentage of CD19^+^ B220^+^ CD27^+^ activated B cells in the spleens of vaccinated mice (*p* < 0.01 and *p* < 0.05 for 0.5% and 1% TOS, respectively) (Figure 5c). Interestingly, the percentage of memory B cells was significantly increased in vaccinated mice fed with DON-contaminated diets (*p* < 0.01), but supplementation with 1% TOS in DON-exposed mice reduced the frequency of memory B cells to the values similar to the control groups (Figure 5c).

#### 4.1.4. Dietary TOS Increased the Production of Type-1 Cytokine IFN-γ and Regulatory Cytokine IL-10 from Re-Stimulated Splenocytes of DON-Exposed Mice

**Figure 6:**
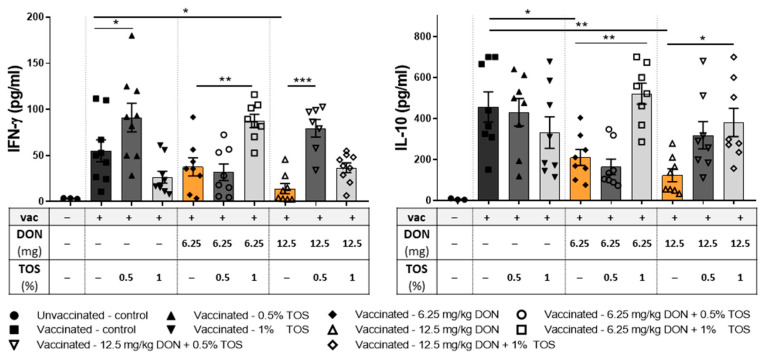
Cytokine production of co-cultured splenocytes of vaccinated (vac +) and unvaccinated (vac −) mice fed on diets containing deoxynivalenol (DON) and/or trans-galacto-oligosaccharides (TOS), with influenza-loaded bone marrow-derived DCs. Interferon (IFN)-γ and interleukin (IL)-10 concentrations in supernatant. Data are presented as mean ± SEM. * *p* < 0.05, ** *p* < 0.01 and *** *p* < 0.001 indicate statistical differences.

In order to study the cytokine production capacity of immunocompetent cells in vaccinated mice, collected splenocytes were re-stimulated ex vivo by co-culturing the cells with antigen-loaded dendritic cells. Dietary TOS or DON did not induce any significant effect on concentrations of IL-4, IL-6, IL-13, TNF-α or MIP-2 in cell supernatants, compared to control-fed vaccinated mice (Supplementary Figure S2). The production of type-1 cytokine IFN-γ and regulatory cytokine IL-10 from splenocytes was significantly lower in vaccinated mice receiving DON in their diet, compared to vaccinated mice receiving the control diet. Dietary supplementation with TOS had no significant effect on the IL-10 release compared to the control diet but significantly increased IFN-γ release from re-stimulated splenocytes compared to the control-fed vaccinated group (*p* < 0.05) and was able to prevent DON-induced reduction in IFN-γ and IL-10 production (Figure 6). Supplementation with 1% TOS significantly increased IL-10 production in mice receiving 6.25 mg and 12.5 mg DON/kg of the diet (*p* < 0.01 and 0.05, respectively), and increased the IFN-γ level in mice receiving 6.25 mg DON/kg of diet (*p* < 0.01). However, a concentration of 0.5% was more effective in preventing the DON-induced IFN-γ reduction in mice receiving 12.5 mg DO/kg in their diet.

#### 4.1.5. Dietary TOS Reversed the Effect of DON on mRNA Expression of Junctional Proteins and Chemokine CXCL1 in the Ileum

**Figure 7:**
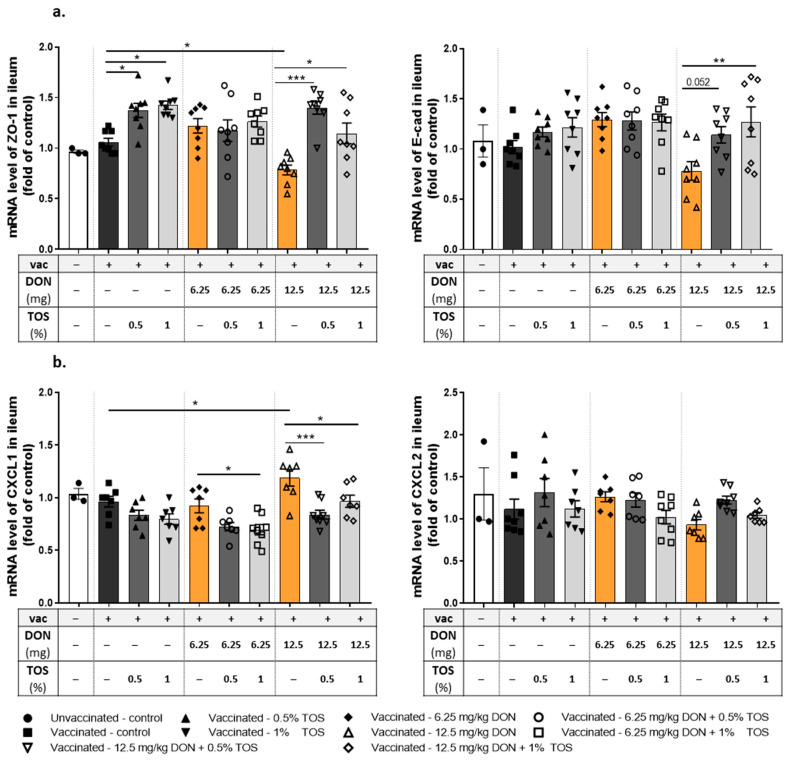
mRNA expression in ileum samples collected from vaccinated (vac +) and unvaccinated (vac −) mice fed on diets containing deoxynivalenol (DON) and/or trans-galacto-oligosaccharides (TOS). Relative mRNA expression of (**a**) tight junction protein zonula occludens (ZO)-1 and adherent junction protein E-cadherin (E-cad), and (**b**) murine chemokines CXCL2 (or MIP-2) and CXCL1 (or KC). Data are presented as mean ± SEM. * *p* < 0.05, ** *p* < 0.01 and *** *p* < 0.001 indicate statistical differences.

The ileal mRNA expression profiles of different junctional proteins were analyzed to evaluate intestinal barrier function, which is known to be affected by DON. Feeding mice with 12.5 mg DON/kg significantly reduced the ZO-1 mRNA expression in the ileum of vaccinated mice. Dietary supplementation with TOS upregulated the expression of ZO-1 mRNA (*p* < 0.05) and reversed DON-induced significant downregulation of this tight junction in the ileum (*p* < 0.001 and *p* < 0.05 for 0.5% and 1% TOS in diets containing 12.5 mg DON/kg) (Figure 7a). Although the effect of TOS or DON on E-cadherin mRNA expression was not statistically significant, the addition of TOS to the diet containing 12.5 mg DON/kg significantly increased the expression of E-cadherin mRNA in the ilea of vaccinated mice (Figure 7b). There was no significant difference between groups in the mRNA expression of OCLD and CLDN-3 proteins (Supplementary Figure S3). Furthermore, mRNA expression of chemokines CXCL2/MIP-2 and CXCL1/KC in ileal sections were measured. These chemokines are regarded as functional homologues of human CXCL8/IL-8, and are thus responsible for the initiation of inflammatory cascades and the recruitment of neutrophils into the mucosa [45]. No significant effect was observed on the expression of CXCL2 by TOS or DON, but ileal mRNA expression of CXCL1 was significantly increased by 12.5 mg DON/kg in the diet in vaccinated mice compared to the control diet (*p* < 0.05), whereas supplementing DON-contaminated diets with TOS prevented the DON-induced upregulation of CXCL1 (Figure 7b).

#### 4.1.6. Dietary TOS Prevented the Adverse Effect of DON on Villus Height of Ileum Sections

**Figure 8:**
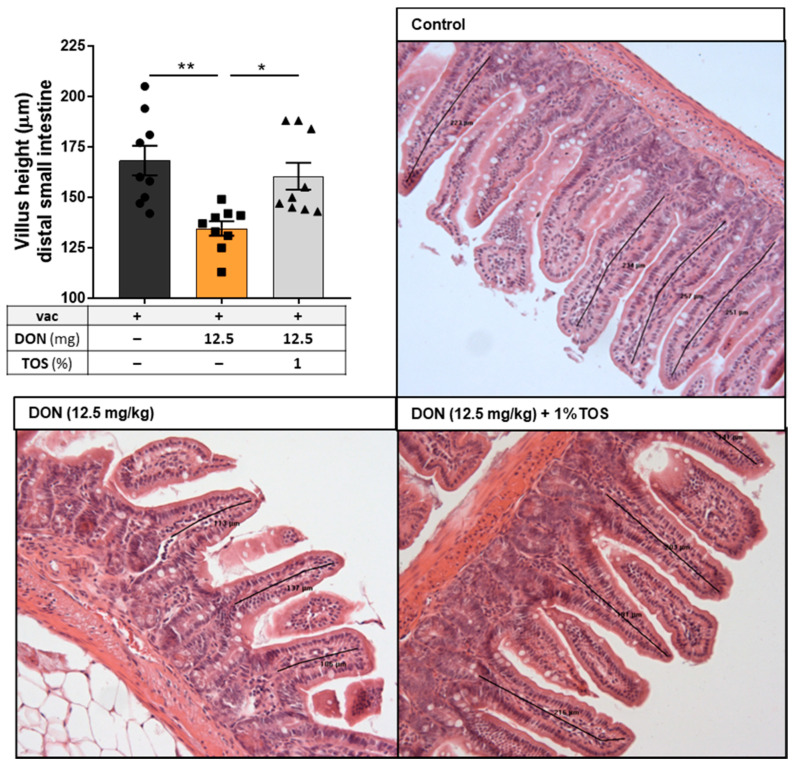
Villus height in ileum sections of the small intestine. Representative photomicrographs of H&E-stained tissue from mice receiving either control or deoxynivalenol (DON, 12.5 mg/kg) ± trans-galacto-oligosaccharides (TOS, 1% wt/wt)-containing diets. Data are presented as mean ± SEM. * *p* < 0.05 and ** *p* < 0.01 indicate statistical differences.

Quantitative histomorphometry analysis was performed on ileum sections of mice fed with control and DON-contaminated diets (12.5 mg DON/kg of diet), with or without 1% TOS. A significant decrease in villus height was observed in the ilea of mice that received 12.5 mg DON/kg in their diet (*p* < 0.01), but the addition of 1% TOS to this diet was able to restore the villus height to normal (Figure 8).

#### 4.1.7. Dietary TOS Had No Significant Effect on SCFA, whereas a Higher Concentration of DON Reduced SCFA Levels in the Cecum

**Figure 9:**
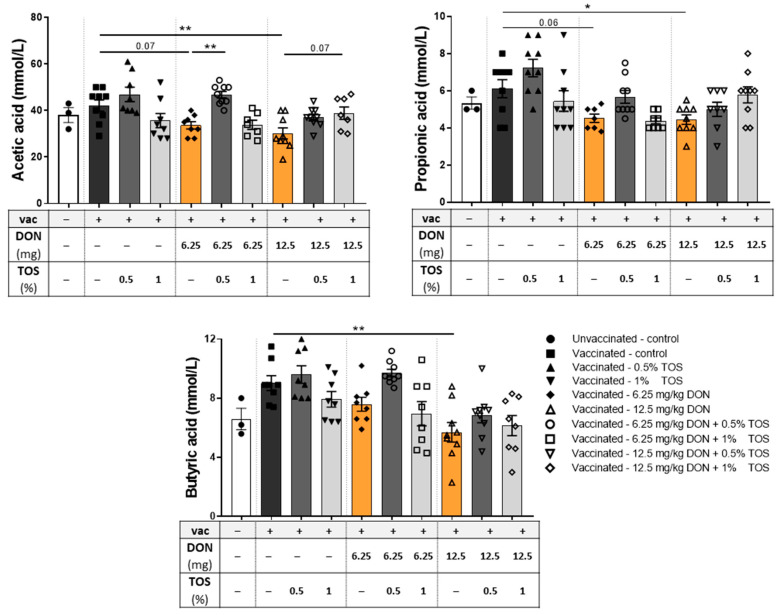
Short-chain fatty acid (SCFA) concentrations in the cecum of vaccinated (vac +) and unvaccinated (vac −) mice fed on diets containing deoxynivalenol (DON) and/or trans-galacto-oligosaccharides (TOS). The concentrations of acetic, propionic and butyric acids were evident in the clear supernatants of cecum contents. Data are presented as mean ± SEM. * *p* < 0.05 and ** *p* < 0.01 indicate statistical differences.

Concentrations of SCFA in the cecum were determined as a measure of microbiota metabolic activity to monitor the changes in the microbiota. Dietary TOS supplementation induced no significant effect on concentrations of different SCFAs in vaccinated mice compared to the control diet. However, DON contamination, especially with higher concentrations, caused a significant reduction in acetate, propionate and butyrate productions in mice undergoing a vaccination-induced DTH reaction (Figure 9). TOS supplementation in DON-contaminated diets significantly elevated the acetate level (*p* < 0.05), but its effect on the DON-induced reduction of propionate and butyrate was not statistically significant (Figure 9).

### 4.2. In Vitro Results: Protective Effect of TOS and Its Main Component, β3′-GL, on DON-Induced Impairment of Caco-2 Cell Monolayer Integrity and IL-8 Production

**Figure 10:**
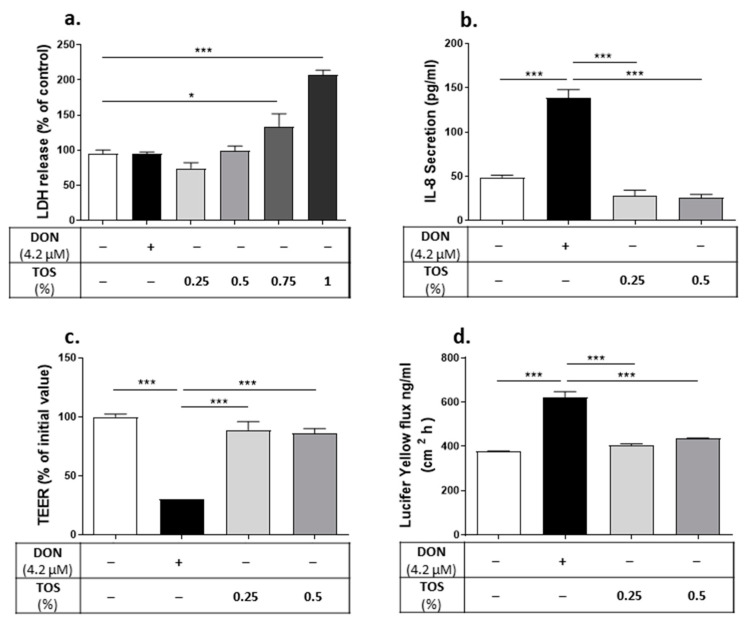
Protective effect of trans-galacto-oligosaccharides (TOS) on deoxynivalenol (DON)-induced adverse effects on Caco-2 cell monolayer. Lactate dehydrogenase (LDH) release in cell supernatant (**a**); IL-8/CXCL8 concentration in cell supernatant (**b**); trans-epithelial electrical resistance (TEER) (**c**) and lucifer yellow flux (**d**). Data are presented as mean ± SEM of 3 independent experiments performed in triplicate. * *p* < 0.05 and *** *p* < 0.001 indicate statistical differences.

The direct cytotoxicity of DON and TOS at tested concentrations were measured via the LDH leakage assay. DON at the concentration of 4.2 µM did not impair cell viability, as indicated by the LDH release in the culture medium (Figure 10a). Treating the cells with TOS at concentrations of 0.25% and 0.5% did not affect the cell viability; however, higher concentrations of TOS (0.75% and 1%) significantly increased the LDH release from Caco-2 cells (*p* < 0.05 and *p* < 0.001, respectively). Therefore, lower concentrations of TOS were used in subsequent experiments.

Pretreatment with 0.25% and 0.5% TOS prevented the DON-induced epithelial barrier disruption, as observed by the increase in TEER values and the decrease in the paracellular flux of LY in DON-exposed cells (Figure 10c,d). Moreover, the DON-induced increase in CXCL8 secretion was prevented by preincubation with both 0.25% and 0.5% TOS (Figure 10b).

**Figure 11:**
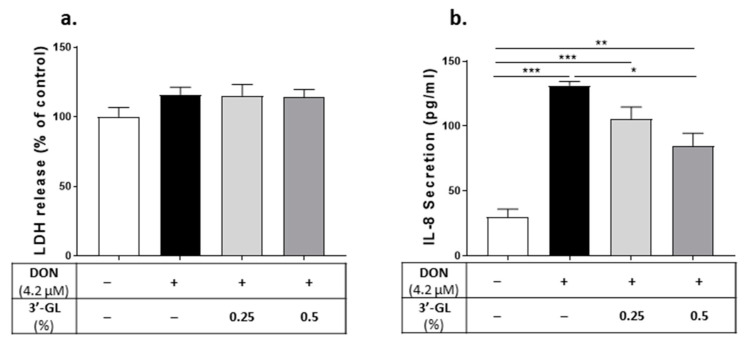

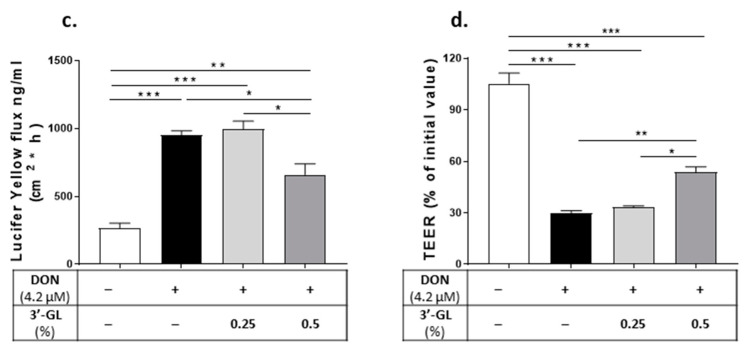
Protective effect of 3′- galactosyllactose (GL) on deoxynivalenol (DON)-induced adverse effects on Caco-2 cell monolayer. Lactate dehydrogenase (LDH) release in cell supernatant (**a**); IL-8/CXCL8 concentration in cell supernatant (**b**); trans-epithelial electrical resistance (TEER) (**c**); and lucifer yellow flux (**d**); Data are presented as mean ± SEM of 3 independent experiments performed in triplicate. * *p* < 0.05, ** *p* < 0.01 and *** *p* < 0.001 indicate statistical differences.

Since the main content of TOS is 3′-GL, protective effects of pure 3′-GLs on DON-exposed Caco-2 cells were studied. Treating Caco-2 cells with 0.25% of 3′GL did not affect the DON-induced barrier disruption and CXCL8 production (Figure 11); however, 0.5% 3′-GL could significantly reduce the DON-induced CXCL8 release (Figure 11b) and improve epithelial barrier integrity in DON-exposed cells, as measured through the reduction in the LY flux and the increase in the TEER value (Figure 11c,d).

## 5. Discussion

Exposure to many food contaminants, such as mycotoxins, is associated with a wide spectrum of effects on the immune system [46]. Given their high prevalence and stability throughout food-processing steps, understanding the immunotoxicity of these food-associated contaminants is crucial in order to come up with efficient preventive strategies. Here, we investigated the adverse effects of the highly prevalent mycotoxin DON on cellular and humoral immune responses to vaccination against the influenza virus in mice, and showed that dietary TOS, a specific mixture of NDOs containing high levels of GL structures similar to those isolated from human milk, induces immune-boosting effects and protects against DON-induced intestinal and immune compromising effects, both in vitro and in vivo.

Dietary supplementation with 0.5% TOS enhanced Th1-dependent cellular and humoral immunity, as measured through increased DTH response and IgG2a production after vaccination. These data are in line with the higher percentage of activated CD69^+^ Th1 cells in the spleen and the increase in the production of IFN-γ from re-stimulated splenocytes of vaccinated mice. Previous studies using dietary intervention with scGOS/lcFOS-containing NDO mixtures [24,31,32], as well as human milk oligosaccharide 2′-fucosyllactose (2′FL) [38] and the combination thereof [47], have shown similar effects on Th1-dependent responses to an influenza vaccine, and increased proliferation of vaccine-specific CD4^+^ and CD8^+^ T cells and higher production of IFN-γ after ex-vivo re-stimulation of splenocytes in vaccinated mice [38]. Different optimal levels of specific NDOs have been observed within this model before. Xiao et al. showed that dietary supplementation with 2′-FL up to 1% increased the influenza-specific DTH response in vaccinated mice dose-dependently, whereas higher concentrations of 2′-FL (>1%) did not show the same pattern [38]. In addition, Vos et al. showed that a dietary intervention with scGOS/lcFOS at a concentration of 5% increased the DTH response, whereas higher concentrations did not induce the same effect in a similar model [32]. This clearly indicates that these oligosaccharides can be the most effective at a certain structure-specific optimum level in this model. Enhancing systemic Th1-dependent adaptive immune responses would lead, in theory, to better immune responses against infections and can be beneficial in attenuating the excessive Th2 responses, which occur in allergies. Such effects have been observed for dietary scGOS/lcFOS and 2′FL, as they could reduce the symptoms of allergic asthma and food allergy in mouse models via the induction of IL-10^+^ T regulatory cells and modulating the Th1/Th2 balance and suppressing Th2-related parameters [48,49].

Adding DON to the diet of vaccinated mice had a specifically detrimental effect on B-cell-mediated humoral immunity, as indicated by reduced vaccine-specific immunoglobulin production. Although supplementation with TOS at a concentration of 0.5% could effectively improve vaccine-specific DTH and IgG levels, a higher concentration of TOS was required to impose similar effects when animals were fed with DON-contaminated diets. DON exposure also decreased Tbet^+^ Th1 cells in the spleen and induced a significant reduction in IFN-γ secretion from splenocytes after ex vivo re-stimulation. These results are in line with previous observations, where compromised resistance to enteric and pulmonary reovirus infections were reported after DON exposure in mouse models [50,51]. DON exposure transiently diminished the host response to reovirus by suppressing IFN-γ and increasing IL-4 mRNA expression in Payer’s patches and consequently suppressing type-1 IFN-mediated responses [50,51]. The results of our study showed that the addition of 1% TOS in DON-contaminated diets increased the frequency of Tbet^+^ Th1 cells and the secretion of IFN-γ from re-stimulated splenocytes, and therefore prevented the DON-induced reduction in type-1 immune responses in vaccinated animals. Moreover, consuming DON-contaminated diets caused a significant drop in the frequency of B cells in the spleen of vaccinated mice, which corresponds to the reduction in vaccine-specific IgG production in these animals. Interestingly, DON-exposed mice had more CD27^+^ memory B cells in their spleens, which could be a compensatory response to the reduced antibody production capacity of these cells. The addition of 1% TOS to the diet of DON-exposed mice restored the percentage of B cells to the values of the control group. It can be concluded that TOS supplementation could restore the Th1/Th2 balance, as well as B cells’ activity in the spleen and improve vaccination responsiveness in DON-exposed mice. 

CD25^+^ and IL-10^+^ regulatory T cells have a prominent role in the immunomodulatory properties of some scGOS/lcFOS-containing NDO mixtures, and some HMOs such as 2′-FL and 6′-sialyllactose (6′-SL) are shown to enhance Th1 and diminish Th2 responses [24,49,52]. Although the presence of TOS alone in mouse diets had no significant effect on regulatory T cells, the addition of TOS to DON-contaminated diets significantly increased CD25^+^FoxP3^+^ Treg cells in the spleens of vaccinated mice. Moreover, dietary TOS increased the regulatory cytokine IL-10 release from the splenocytes of DON-exposed mice. The observed effects of TOS on regulatory T cells and IL-10 could be a reaction to the Th2-skewing effects of DON and could represent a mechanism to restore the Th1/Th2 balance after DON exposure. 

Another possible explanation for the immunomodulatory properties of prebiotic oligosaccharides may be that they affect microbiota-dependent mechanisms by rebalancing microbiota composition in the gut [25]. There is a potential link between changes in gut microbial metabolites and improved vaccine-specific immune responses upon HMOS supplementation [47,53]. Clinical analysis of SCFA concentrations in fecal samples of 3–5 months old infants showed that in exclusively breastfed infants the relative proportion of acetate was higher compared to non-breastfed infants [54]. SCFA profiles produced upon fermentation of HMOs by gut bacteria have well-established anti-inflammatory properties and regulate innate immune cells, such as macrophages, neutrophils and DCs, as well as antigen-specific adaptive immunity, mediated by T cells and B cells [55]. SCFAs have a significant impact on regulatory T cells and effector T cells by upregulating gene expression during lymphocyte activation and can enhance the mucosal and systemic antibody responses [55]. DON-contaminated diets significantly reduced SCFA concentrations in the cecum content of mice, and this effect was restored through the addition of TOS to the diets. The effect of TOS on SCFA was more prominent in terms of the acetic acid concentration, indicating that TOS enhances specific microbial communities in the gut. Acetate is produced in high levels by certain bacteria, such as various *Bifidobacterium* and *Bacteroides* species, and can enhance mucus production and goblet cell differentiation, thereby supporting the intestinal epithelial barrier and immune function [56]. Moreover, *Bifidobacteria* strains in the human gut are able to utilize 3′-GL efficiently [57]. The prebiotic properties of TOS possibly play an important role in modulating immune responses.

DON is known to disrupt epithelial barrier function and integrity, mainly by disrupting the expression and localization of junctional proteins [12,58], and it can reduce the villus height in the intestine [12]. In the present study, feeding mice diets contaminated with DON suppressed the mRNA expression of tight junction protein ZO-1 in the ileum, and shortened the height of the villi, which is in line with the observations in previous studies. Shortened villus height could possibly be part of the repair mechanism to overcome the barrier dysfunction after DON exposure by reducing the surface area of the villi [59]. The addition of TOS to DON-contaminated diets upregulated mRNA expression of junctional proteins and restored the villus height in ileal sections to control values in vaccinated mice. Similar beneficial effects have been reported for scGOS as it can attenuate the destructive effects of DON on villus architecture in B6C3F1 mice, and the intestinal barrier of Caco-2 cells, possibly by stimulating the tight junction assembly in the epithelial cell layer [13]. Similarly, administration of 2′-FL in rats had a trophic effect, indicated by higher villus heights and areas in the intestine [60]. The observed effect of TOS could be a result of directly enhancing epithelial barrier maturation and mucus production and modulating the expression of junctional proteins [61], or could be due to the enhanced production of SCFAs, which are known to improve intestinal barrier integrity and protect the mucosal layer [62,63].

Imbalanced expression of junctional proteins leads to a leaky intestinal barrier and allows antigens to cross the epithelium more easily, resulting in the production of cytokines. This can influence the uptake and processing of foreign antigens by DCs, and therefore influence the development of effector cells from naïve T cells [20]. Murine chemokines CXCL2/MIP-2 and CXCL1/KC are known to be functional homologs of human CXCL8 and have been found to contribute to the initiation of inflammatory cascades and the recruitment of neutrophils into the mucosa [45]. In the present study, DON exposure increased the expression of chemokine CXCL1/KC in ileal sections, which was significantly suppressed by dietary TOS supplementation. Similar anti-inflammatory properties and reductions in CXCL1/LC expression have been reported for scGOS in DON-exposed mice [13]. The release and synthesis of these chemokines are Toll-like receptor (TLR)4-dependent and it is known that intestinal epithelial cells are capable of expressing this receptor [64]. There is a significant role for TLR4 in the immunomodulatory properties of different HMOs, such as 2′-FL, and NDO mixtures of scGOS/lcFOS [20,65]. Therefore, TLR4-dependent pathways may possibly be partly involved in the anti-inflammatory effects of TOS in the intestine. Further investigations are required to fully understand the mechanisms involved in the protective effects of TOS against DON-induced intestinal barrier disruption and inflammation.

To confirm the in vivo observations on intestinal integrity and to study the direct effect of TOS and its main component, 3′-GL, on the epithelial barrier in more detail, single-cell monolayers of the Caco-2 cell line were used as an in vitro model of the human intestinal epithelial barrier. Caco-2 cells are able to fully polarize to form brush borders and cell–cell junctions, and therefore represent the morphologic characteristics of normal human enterocytes [66]. Pre-treatment with TOS prevented the adverse effect of DON on the barrier integrity of the Caco-2 cell layer, and reduced DON-induced CXCL8 production. These results are in line with the in vivo observations in this study and confirm the protective effect of TOS on the intestinal epithelial layer. Similar beneficial effects were observed for 3′-GL on DON-induced barrier disruption and CXCL8 production. However, the observed effects on the Caco-2 cell model cannot be explained by TLR4-dependent pathways, since these cells have very low surface expression of TLR4 [67], indicating that other pathways might be involved in the protective properties of 3′-GL on the intestine.

In conclusion, exposure to DON downregulates immune responses to vaccination through reducing Th1-mediated cellular and humoral immune responses in mice. Dietary intervention with specific oligosaccharides, TOS, can attenuate the adverse effects of DON on the systemic adaptive immune response by restoring the Th1/Th2 balance and improving vaccine responsiveness. It can also re-establish gut microbial activity and protect the intestinal epithelial barrier. The results of in vitro experiments indicate that the observed properties of TOS on gut microbiota and the epithelial barrier are possibly linked to the 3′-GL present in the mixture, which is one of the specific oligosaccharide structures present in human milk. Therefore, dietary supplementation of infant formulae with TOS could be beneficial in boosting the immune system and in the development of a healthy gut microbiome and preventing the harmful effects of food-contaminant DON on the intestinal and immune systems. We also showed that 3′-GL, the main component of TOS, is in part responsible for the observed effects of TOS on the intestine, which signifies the importance of studying the effects of individual oligosaccharide structures on intestine and systemic immune development.

## Figures and Tables

**Table 1:** Experimental groups, and concentrations of deoxynivalenol (DON) and trans-galacto-oligosaccharides (TOS) in the diets.

Dietary Groups	Vaccination	*n*
Control (AIN93G)	-	3
Control (AIN93G)	+	9
0.5% TOS (wt/wt)	+	9
1% TOS (wt/wt)	+	9
DON (6.25 mg/kg of diet)	+	9
DON (12.5 mg/kg of diet)	+	9
DON (6.25 mg/kg) + 0.5% TOS	+	9
DON (12.5 mg/kg) + 0.5% TOS	+	9
DON (6.25 mg/kg) + 1% TOS	+	9
DON (12.5 mg/kg) + 1% TOS	+	9

## Data Availability

The original contributions presented in the study are included in the article and Supplementary Materials. Further inquiries can be directed to the corresponding authors.

## Supplementary Materials

The following are available online at https://www.mdpi.com/article/10.3390/nu13093190/s1, Figure S1: HPAEC-PAD generated TOS fingerprint (**a**), GOS (**b**), and Beta1,3-galactosyllactose standard (3’-GL) (**c**). Figure S2: Cytokines production of co-cultured splenocytes with influenza-loaded bone marrow-derived 10 DCs. Interleukin (IL)-4, IL-6, IL-13, tumor necrosis factor (TNF)-α and macrophage inflammatory protein (MIP)-2 concentrations in culture supernatant. Figure S3: Relative mRNA expression of tight junction proteins occludin (OCLD) and claudin-3 (CLDN-3)in ileum samples collected at day 31. Data are presented as mean ± SEM. Table S1: List of antibodies used in flowcytometry analysis. Table S2: List of relevant comparisons implemented in statistical analysis.
