# Supplementary material for: Human Milk Oligosaccharide 3′-GL Improves Influenza-Specific Vaccination Responsiveness and Immunity after Deoxynivalenol Exposure in Preclinical Models
Source: Nutrients. 2021 Sep 14;13(9):3190. doi: 10.3390/nu13093190 (PMC8466816; doi:10.3390/nu13093190)
Supplement: Supplementary file 1 [file nutrients-13-03190-s001.zip › nutrients-1363394-supplementary.pdf]

Supplementary content:

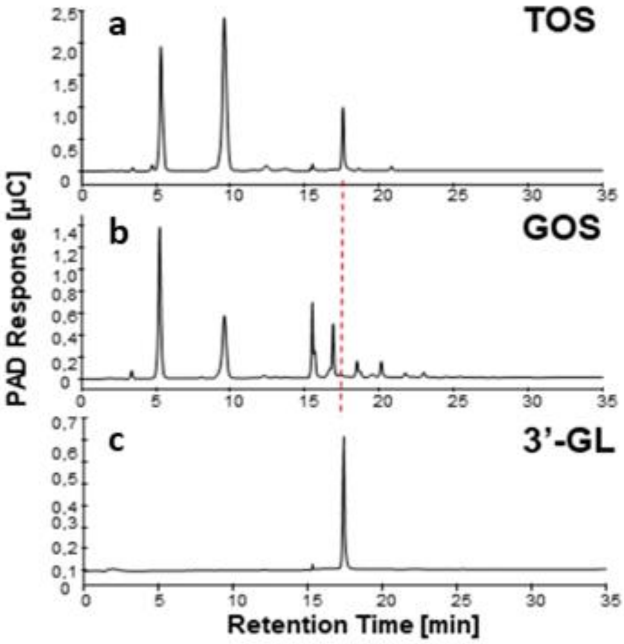

Supplementary Figure S1. HPAEC-PAD generated TOS fingerprint (a), GOS (b), and Beta1,3-galactosyllactose standard (3'-GL) (c).

Supplementary Table S1. List of antibodies used in flowcytometry analysis.

Marker	Label	Catalog number	Antibody ID	Dilution
CD4	BV510	100553	AB_2561388	1:160
CD69	PE-Cy7	25-0691	AB_469637	1:640
T1/ST2	FITC	101001F	AB_947549	1:200
CXCR3	PE	12-1831	AB_1210734	1:100
Tbet	Alexa Fluor647	644803	AB_1595573	1:1600
Gata3	PerCP-eFluor710	46-9966	AB_10804487	1:1000
CD25	PerCP-Cy5.5	45-0251	AB_914324	1:1200
CD196 (CCR6)	PE	129804	AB_1279137	1:640
FoxP3	FITC	11-5773	AB_465243	1:100
RorgT	Alexa Fluor647	562682	AB_2687546	1:400
AHR	PE-Cy7	25-5925	AB_2573501	1:200
CD3	Percp-cy5.5	45-0031	AB_1107000	1:100
CD19	APC	17-0193	AB_1659676	1:75
B220	FITC	11-0452	AB_465054	1:75
CD27	PE	12-0271	AB_465614	1:75

Supplementary Table S2. List of relevant comparisons implemented in statistical analysis.

Sham-treated group	vs.	Control (vaccinated)
Control (vaccinated)	vs.	0.5% TOS (wt/wt)
		1% TOS (wt/wt)
		DON (6.25 mg/kg of diet)
		DON (12.5 mg/kg of diet)
DON (6.25 mg/kg of diet)	vs.	DON (6.25 mg/kg) + 0.5% TOS
		DON (6.25 mg/kg) + 1% TOS
DON (12.5 mg/kg of diet)	vs.	DON (12.5 mg/kg) +0.5% TOS
		DON (12.5 mg/kg) + 1% TOS

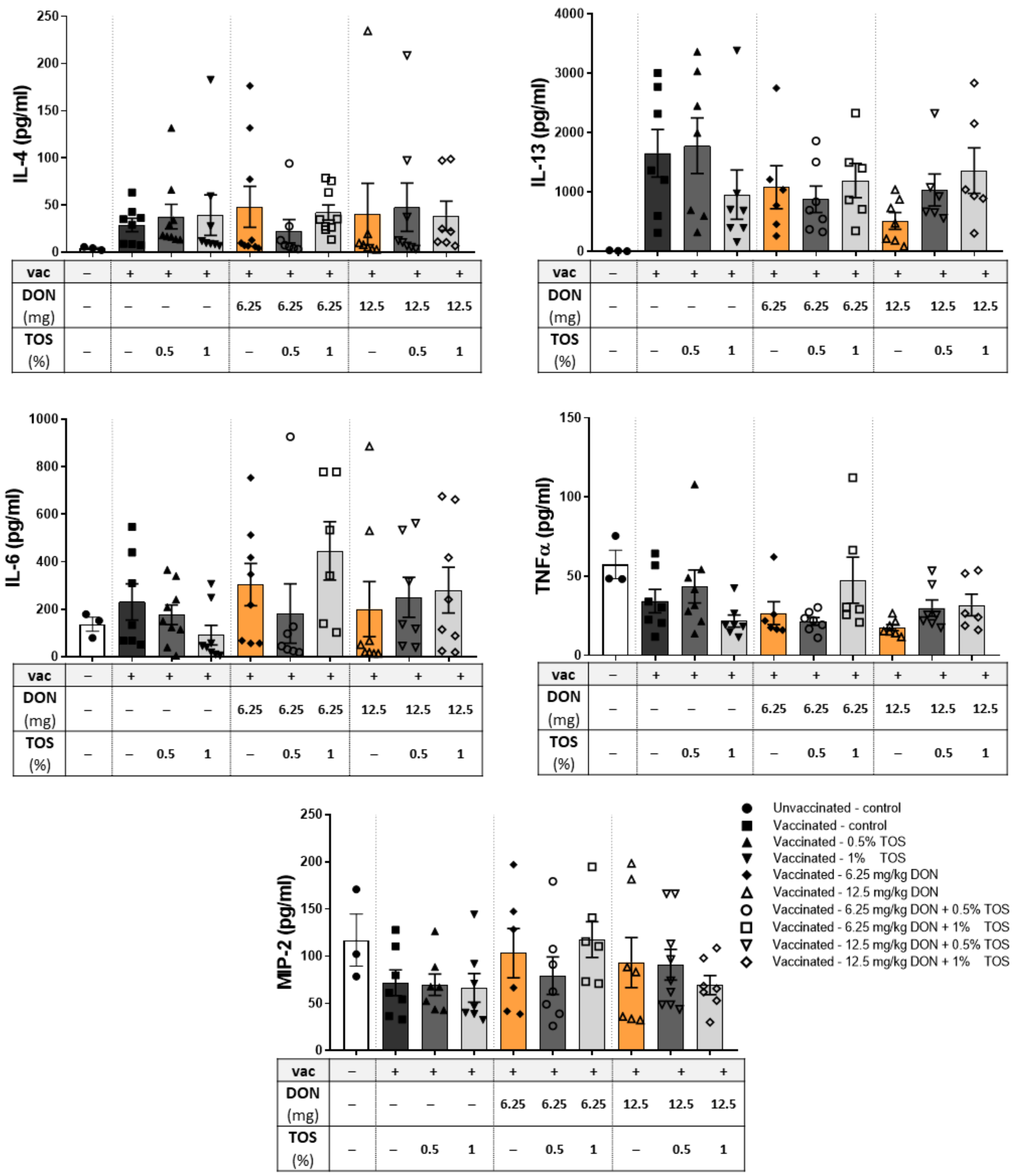

Supplementary Figure S2. Cytokines production of co-cultured splenocytes with influenza-loaded bone marrow-derived DCs. Interleukin (IL)-4, IL-6, IL-13, tumor necrosis factor (TNF)-α and macrophage inflammatory protein (MIP)-2 concentrations in culture supernatant.

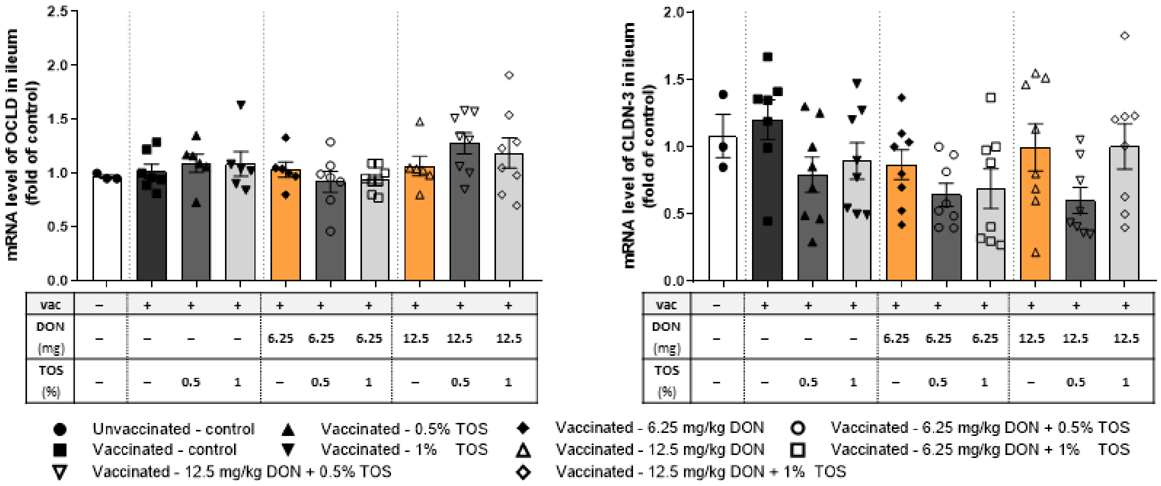

Supplementary Figure S3. Relative mRNA expression of tight junction proteins occludin (OCLD) and claudin-3 (CLDN-3)in ileum samples collected at day 31. Data are presented as mean ± SEM.
